# Autogenerating a Domain-Specific Question-Answering Data Set from a Thermoelectric Materials Database to Enable High-Performing BERT Models

**DOI:** 10.1021/acs.jcim.5c00840

**Published:** 2025-08-07

**Authors:** Odysseas Sierepeklis, Jacqueline M. Cole

**Affiliations:** † Cavendish Laboratory, 2152 University of Cambridge, J. J. Thomson Avenue, Cambridge CB3 0HE, U.K.; ‡ Science and Technology Facilities Council, Rutherford Appleton Laboratory, Harwell Science and Innovation Campus, Didcot, Oxfordshire OX11 0QX, U.K.

## Abstract

We present a method for autogenerating a large domain-specific question-answering (QA) dataset from a thermoelectric materials database. We show that a small language model, BERT, once fine-tuned on this automatically generated dataset of 99,757 QA pairs about thermoelectric materials, affords better performance in the field of thermoelectric materials compared to a BERT model fine-tuned on the generic English-language QA data set, SQuAD-v2. We further show that mixing the two data sets (ours and SQuAD-v2), which have significantly different syntactic and semantic scopes, allows the BERT model to achieve even better performance. The best-performing BERT model fine-tuned on the mixed data set outperforms the models fine-tuned on the other two data sets by scoring an exact match of 67.93% and an *F*1 score of 72.29% when evaluated on our test data set. This has important implications as it demonstrates the ability to realize high-performing small language models, with modest computational resources, empowered by domain-specific materials data sets which can be generated according to our method.

## Graphical abstract

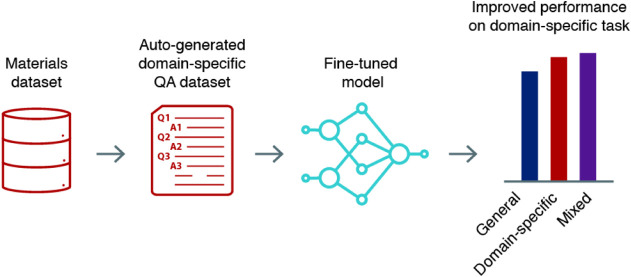

## Introduction

Discriminative language models are typically pretrained on a generalized corpus and then fine-tuned on a labeled data set for bespoke tasks. Recently, language models for the materials-science domain have either been pretrained from scratch, or further pretrained from their original weights, using domain-specific corpora as input to improve model performance on downstream tasks.^1–6^ One of the most highly desirable tasks for language models in materials science is relationship extraction, which can be framed as a multiturn extractive question-answering (QA) task.^7^ Thereby, material and property information can be extracted from literature, using a language model that has been fine-tuned on a carefully sequenced set of questions and answers, such as

Question 1 - “What is the material?” (example answer: “water”),

Question 2 - “What is the boiling point of < the answer to the previous question>?” (example answer: “100^◦^C”).

This conditional style of questioning draws out material-property paired quantities from the literature that researchers can use to identify structure–property relationships in the given domain. These relationships can then be incorporated into a chemical design process that aims to accelerate prediction and discovery of materials tailored to the desired application.

Several attempts to extract material-property databases from large language models have been reported recently.^8–11^ However, such efforts have tended to employ foundational large language models and have used interactive human prompting or have been fine-tuned for QA tasks using large nondomain specific data sets of question and answer pairings, such as the Stanford Question Answering Data set (SQuAD),^12^ with 100,000 pairs. A few small QA data sets of materials science applications have been reported.^4,5,13^

Nonetheless, the research community lacks large data sets of QA pairings that are specific to a given domain of materials science; even though they would be extremely useful by employing them to fine-tune a language model to deliver significantly more informative data-extraction performance.

Li and Cole^14^ recently presented a solution to this issue: by combining the Chemical Named Entity Recognition (CNER) strengths of ChemDataExtractor to extract material-property paired quantities, with new algorithms that employ these quantities to autogenerate materials-domain-specific QA data sets. An algorithm first identifies the text spans in a corpus from which the material-property data in a given ChemDataExtractor-generated materials database had been sourced; then another algorithm employs this material-property information from the materials database, and the source text spans, to automatically produce a large data set of QA pairs. This large domain-specific question-answering data set can then be used to fine-tune a foundational language model for downstream QA tasks that are geared to a certain materials-science-specific domain of interest.

Such language models can be relatively small given that the power of the materials-domain knowledge rests in the large QA data set, i.e., outside the language model. This is important because it means that vanilla small language models (SLMs) can simply be fine-tuned with materials-domain specific QA data sets to deliver high performance in a targeted domain of interest; such a procedure requires modest computational resources, thereby ensuring computational speed and energy sustainability, and is relatively simple to perform.

Li and Cole demonstrated the power of their approach using case studies within the field of photovoltaics.^15^ The study presented herein explores their approach within another field of energy research: thermoelectrics. The main avenue of investigation is the potential of transforming an existing ChemDataExtractor-generated thermoelectric materials database into a bespoke QA data set about thermoelectric materials and properties to bolster the performance of materials-property data-extraction tasks that operate on an SLM. Moreover, the study examined the potential of combining generic English-language QA data sets, such as the well-established SQuAD-v2 ^16^ with this narrow domain-specific data set to further improve extractive performance. The idea behind this QA data set mixing is that the generic database can “teach” the model the general syntax of a target language (in this case, English), while the material-domain-specific data set can inform the model about the semantics of the scientific field. The process of algorithmically creating a large QA data set from an existing materials-science database is presented, as well as a performance comparison of SLMs that have been fine-tuned on these different QA data sets, by testing them on the task of information extraction for the thermoelectric materials domain. This study differs from the study of Li and Cole in terms of: 1) the question-forming approach (this study also generates unanswerable questions); 2) the model fine-tuning process (we use hyperparameter optimization with a fixed model, while Li and Cole examined a wider variety of variables, such as model size, data set size, and different cased model variations); 3) an investigation of combining the two QA data sets with different merits (undertaken in this study); and 4) the type of test data set on which the fine-tuned models were evaluated (ours was manually annotated, as opposed to being automatically generated as in the Li and Cole study).

Bidirectional Encoder Representations from Transformers (BERT) models were our language models of choice in this study because BERT models are open-source and their continuous developments are community led; as such, a range of good benchmarks of materials-science-based BERT models exist^4–6^ against which we can readily compare our performance results. Given the massive size upscaling of language models that the AI field has witnessed since the original BERT model was released, we herein classify our BERT models as SLMs rather than their original description as large language models (LLMs). This work used the publicly available BERT-base-uncased checkpoint (12 transformer layers, hidden size 768, 110 M parameters), which was originally pretrained on BookCorpus and English Wikipedia (approximately 3.3 B tokens).^17^ We did not vary model size in this study.

## Methods

### Crafting a Domain-Specific Test Data Set

Figure 1 depicts the workflow followed in this project. The first step of the process was to create a QA data set on which the language models would be tested. An assortment of 281 paragraphs with extractable full data records was randomly sampled from approximately 60,000 articles that had been downloaded from Elsevier, Springer, and the Royal Society of Chemistry (RSC) publishers during the course of previous work.^18^ A *full record* refers to text spans within a context which defines the following: a chemical compound; one of the seminal thermoelectric materials properties (*ZT*, the Seebeck coefficient, thermal conductivity, electrical conductivity, power factor); a numerical value for that property—with its units, if applicable; and the temperature at which the value was recorded. The temperature can be identified either qualitatively (e.g., “room temperature”) or quantitatively, comprising a numerical value and the associated units. Each question in the data set targets one of these components of a full record.

**Figure 1:**
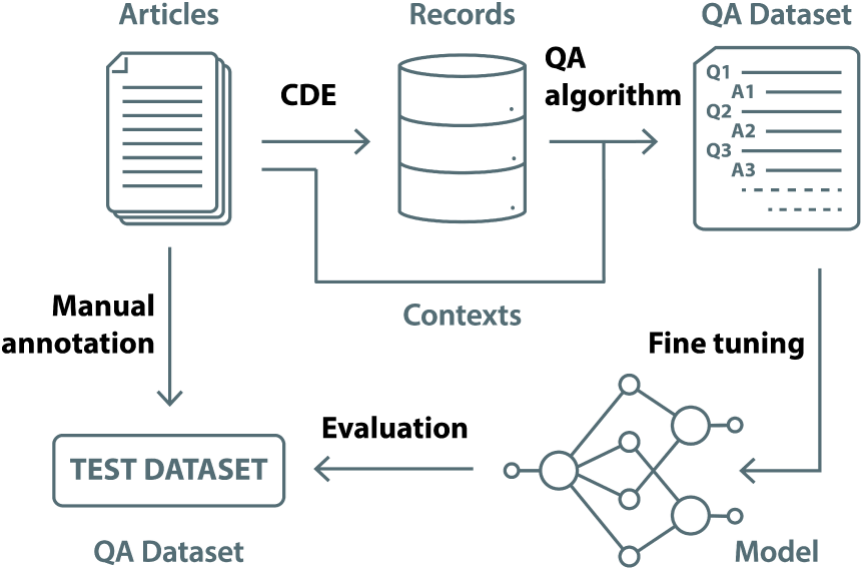
Schematic of the operational pipeline developed and applied in this study.

The web-based annotation tool, BRAT,^19^ was used to expedite the creation of this test data set. BRAT stands for BRAT Rapid Annotation Tool and provides functionality that facilitates the manual annotation of text documents. There exists a plethora of annotation tools^20^ which could have been used for this part of the process, but BRAT was identified as being straightforward and sufficiently versatile for the desired task. First, a BRAT annotation configuration was defined which reflects the relationships between material, property specifier, property reading, and temperature, which encapsulate a full thermoelectric record. 603 data records were manually annotated from the 281 randomly sampled paragraph contexts. An example of the BRAT annotation interface, with context from the thermoelectric materials domain, can be seen in Figure 2. BRAT stores information on each annotation locally and code was then written to transform each annotation into a set of QA pairs, similar to the algorithm which will be described in the following subsection. The QA data sets generated in this report adhere to the structure and logic of SQuAD-v2,^16^ which includes both answerable and unanswerable questions from the given context. The inclusion of unanswerable questions in this work is motivated by the fact that in most practical use cases, the ability of a model to distinguish whether or not an answer exists in the context is highly desirable, to avoid the extraction of untrue results. This process generated 2,722 QA pairs from the manually labeled contexts, of which 1,809 were answerable questions, and 913 were unanswerable. This QA data set, used for testing, will be referred to as the thermoelectric materials QA *test data set* during this project, to distinguish between itself and the validation sets used during the hyperparameter optimization sweeps. The BRAT manual annotation files, the thermoelectric QA test data set, as well as the code that converts the local annotation files to QA pairs have been made available online.^21–23^

**Figure 2:**
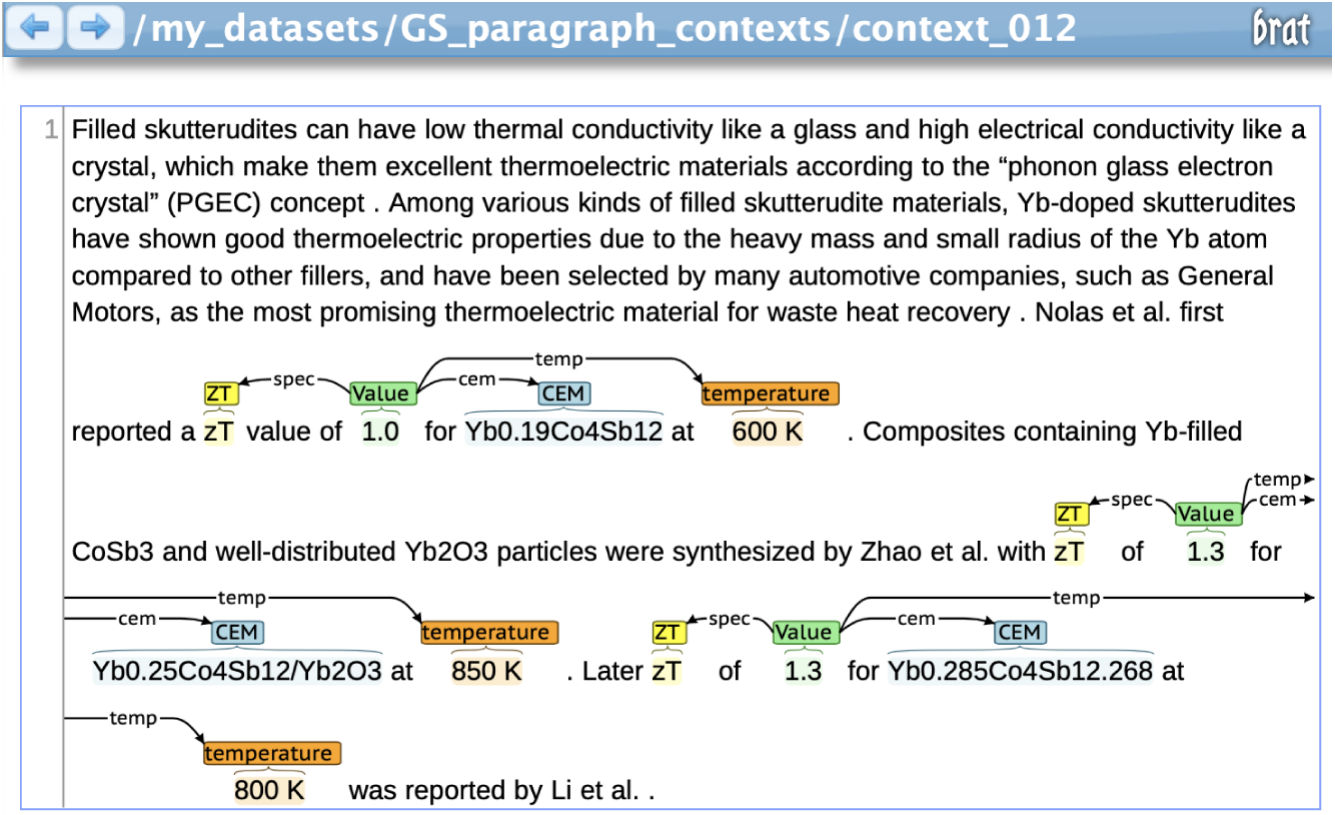
Example of three annotated thermoelectric material *ZT* data records using the BRAT rapid annotation tool.^19^ Screenshot reflects original annotations on text reproduced under the terms of the Creative Commons CC BY-NC-ND license from ref.^24^ Tang et al. 2015, Elsevier.

### Creating a QA Data Set from a Materials-Science Database

Generating a QA data set from a materials-science database follows a similar algorithmic method to the one used by Li and Cole,^14^ based on the prospect of framing an entity-relation extraction task as a multiturn QA task^7^ and applying this to an existing materials database. The database used for this paper was a database of thermoelectric materials that had previously been automatically extracted from the scientific literature at the sentence level.^18^ The extractions were performed using the chemistry-aware natural-language-processing toolkit, ChemDataExtractor^25,26^ adapted for application in the thermoelectric materials domain. The database consists of 22,805 data records that span 10,641 unique extracted chemical names. Each data record holds the value of one of the aforementioned seminal thermoelectric properties, along with the temperature of the material that was recorded during property measurement, as well as some other optional complementary information such as pressure during a measurement or the direction of a measurement. The database was publicly released to facilitate data-driven materials discovery in the thermoelectric materials domain.^27–29^ Figure 3 illustrates the percentages and absolute numbers of the extracted data records for the different thermoelectric properties in this database. Table 1 shows the precision, recall, and number of data records for the automated retrieval performance metrics of the database as a whole, as well as for each extracted property in the database.

**Figure 3:**
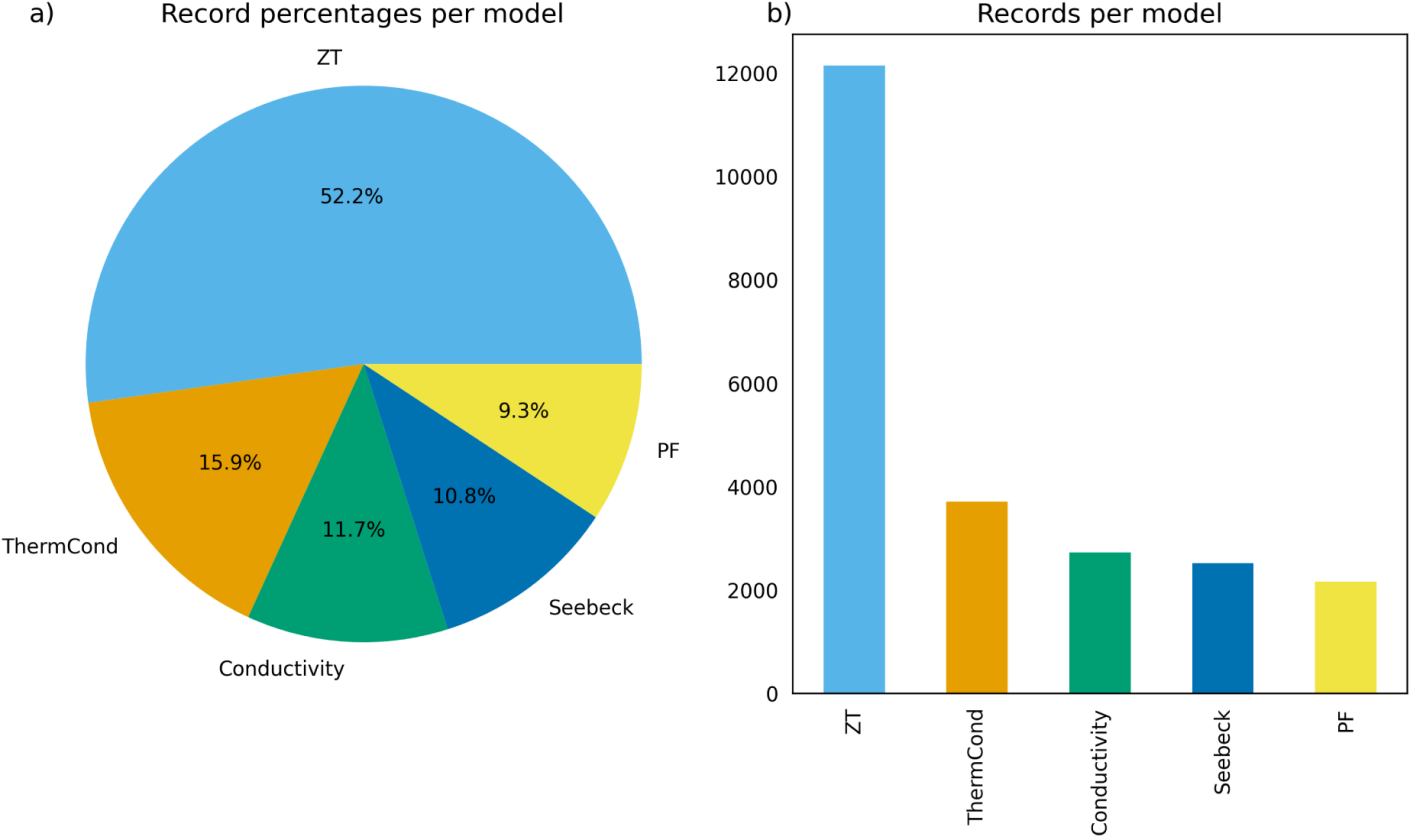
Percentages (a) and absolute number (b) of data records per property found in the thermoelectric materials database^18^ used to generate the QA data set.

**Table 1:** Precision, Recall, and Number of Data Records for Each Model, Extracted with Temperature

Model	Precision (%)	Recall (%)	Number of data records
*ZT*	83.00	43.71	11668
Thermal conductivity	83.50	37.00	3701
Seebeck coefficient	78.50	33.00	2584
Electrical conductivity	78.50	34.97	2728
Power factor	85.0	31.18	2224
Weighted Average	82.25	39.23	N.A.

We developed an algorithm that transforms data records found in this database into a set of QA pairs. The QA pairs result from a multiturn QA framing of an entity-relation extraction task. Thereby, the sentences that were used to construct each record in the database were first recovered so that they could be used as contexts for the questions and answers that the algorithm generates. The questions start from the property value and units found in the sentence context and build up to the next data record by iteratively asking and answering a tuple of conditional questions, as illustrated in Figure 4. This allows each new question to use the answer of the previous one to build up to the full record, starting from the property value and querying for the associated temperature reading, then the property specifier, and finally the material to which these readings pertain. This bottom-up approach, starting from the value and units (measurement) identified in the originating literature text and building up to the their associated material identity, may offer some practical benefits: first, it is more common for one material to be associated with multiple properties and measurements, rather than the other way around. The latter option of asking a question starting from the material might have multiple correct answers, which necessitates identifying those answers and then asking different questions based on that information to complete a record. This problem is less likely to occur when starting from the individual property measurements, as per our methodology. Second, while toolkits such as ChemDataExtractor have demonstrated improved performance in CNER, such recognition still remains a challenging task for specialized domains, while it is considerably easier to define rules which extract values and units from a field.^18,26^ Our procedure affords three different QA pairs per unique data record in the database. While the questions are generated in a sequential order, with each question in the set depending on the answer of the previous one, they are entered in the data set independently. This allows the model to learn each question on its own, as if it always returns the previous answers correctly, which is akin to the teacher-forcing technique.^30^ In many cases, databases such as the ones generated using ChemDataExtractor^18,31–37^ might involve formatting the material name or other entries. Our QA-data set-generating algorithm further attempts to check various possible renditions of the entries, recovering characters such as whitespace or hyphens in order to ensure that the answers can indeed be identified in the sentence context.

**Figure 4:**
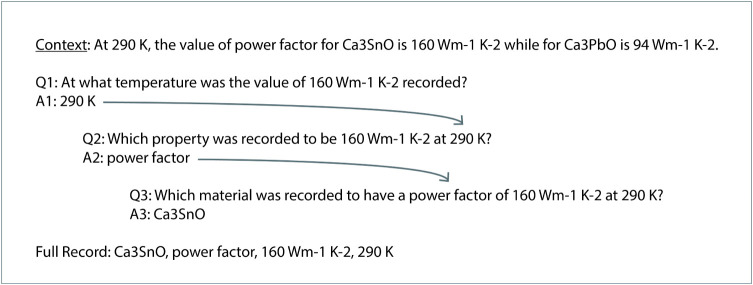
An example of how previous answers from the sentence context cascade into the following questions in order to frame an entity-relation extraction task.

Another important facet of our algorithm is its ability to generate unanswerable questions from the given context. This happens by picking an “unfindable data point” when asking a question. This data point is randomly sampled from the pool of entries found in the thermoelectric materials database. By taking into consideration the type of data point that is being sought, appropriate rules were defined to randomly select a data point that renders the question unanswerable from the given context. The rules ensure that the unfindable data point is of the same type as the type being sought (e.g., that a property specifier is replaced by a property specifier, and not, for example, a temperature reading, as that would make the question very obviously unanswerable); that the unfindable data point is not equivalent to the one being replaced (e.g., “*ZT*” should not be replaced by “figure of merit”, since that forms the same question semantically); and that the unfindable data point is indeed not present in the sentence context. More information on the details of this approach and these rules can be found in the commented code of the algorithm. This approach can potentially generate three unanswerable questions per database record, similar to the answerable questions described above. Random sampling was employed throughout the QA data set production in order to remain consistent with the approximate ratio of 2:1 of answerable to unanswerable questions found in the original SQuAD-v2 publication,^16^ and to introduce variety to the number of QA pairs generated per context. This means that while all the possible answerable questions were added to the QA data set, each potentially generated unanswerable question was added according to a 50% chance in order to ensure the desired ratios. This approach afforded a total of 99,757 QA pairs for the generated thermoelectric-specific QA data set. 66,508 of these QA pairs were answerable questions and 33,249 were unanswerable. There were fewer than 5% failed attempts at generating an answerable QA pair from the sentence context, where the recorded answer could not be located therein.

The QA data set generated from this algorithmic procedure will be referred to as the *TE-CDE-QA* data set, since it derives from a thermoelectric (TE) materials database that was autogenerated using ChemDataExtractor. The efficacy of this QA data set was investigated by comparing how well a BERT language model performs after fine-tuning it on this specially crafted data set when evaluated on the thermoelectric test data set. The TE-CDE-QA data set was compared with the generic English-language SQuAD-v2. An important distinction between the two QA data sets, is that the contexts of the TE-CDE-QA data set span only single sentences, while SQuAD-v2 asks questions whose answers can span multiple sentences within a paragraph. This offers SQuAD-v2 syntactic advantage, since the test data set asks from paragraph contexts, while TE-CDE-QA has the semantic advantage of being specialized in the target domain. SQuAD-v2 enjoys a further advantage due to it being a human-crafted data set, where one might assume an accuracy of near 100% in its answers. In contrast, the TE-CDE-QA data set was algorithmically created from a materials database which has been automatically generated using ChemDataExtractor; as such that the accuracy is likely to be somewhat lower, as discussed in the original paper.^18^ A combination of the two QA data sets has also been introduced in our performance comparison, referred to as the *mixed* data set, which is a simple mixing of the two data sets. The mixed data set therefore has 241,949 QA pairs which include both sentence and paragraph-scope contexts. All data sets contain both answerable and unanswerable questions at a ratio of approximately 2:1. The algorithm that generated the TE-CDE-QA data set, the TE-CDE-QA data set itself, and the mixed version between TE-CDE-QA and SQuAD-v2 have been made available online.^23,38^ Table 2 shows the numbers of questions for the three QA data sets used in this project.

**Table 2:** Number of Answerable and Unanswerable Questions per QA Data Set

QA Data set	Total	Answerable	Unanswerable
SQuAD-v2	142,192	92,749	49,443
TE-CDE-QA	99,757	66,508	33,249
Mixed	241,949	159,257	82,692

### Fine-Tuning Language Models with Hyperparameter Optimization on Different QA Data Sets

Language models have been shown to afford better performance on in-domain downstream tasks when pretrained on in-domain data sets.^1–6^ Fine-tuning is the process by which the weights of the last layer(s) of a pretrained model are updated using a labeled data set for a specific task. This allows the language model to adapt to the task seen in the fine-tuning data set, while still leveraging the more computationally intensive weight adaptations achieved during pretraining. The task that was trained via the subject data sets was extractive question-answering, which can be used in a multiturn QA fashion to perform downstream entity-relation extraction from contexts.

Extractive QA using the BERT model is typically achieved by adding a span-classification layer on to the final neural-network layers of the language model. This span-classification layer consists of two components, one which predicts the start index of the answers and the other one which predicts the end index. Each component uses a linear layer that projects its hidden states into different scores for each possible position. The scores are then converted into probabilities which allows the model to return its estimate of the most likely answer. Accordingly, the training, validation, and test data sets used to train the language model include the start and end indices of the desired answers. These indices have been generated by the QA-data set-generating algorithm described in the previous section. Unanswerable questions are typically represented by the two components of the layer, returning a special result, such as a designated token or index out of bounds, which the models are trained to predict. The QA data sets were split using a train-to-validation ratio of 90%:10%. The models which performed the best on their respective validation set were then identified via the chosen evaluation metrics. Finally, those models were evaluated on the aforementioned thermoelectric test data set that we had generated from text annotations using BRAT in order to investigate the efficacy of the different QA data sets.

The main language model used to test the QA data sets was BERT-base,^17^ using the Huggingface transformers library implementation.^39^ We fine-tuned all 12 encoder layers of BERT-base without freezing any parameters. This was possible owing to the availability of tens of thousands of automatically generated QA pairs from our thermoelectric-materials database. A Bayesian optimization technique was employed to explore different hyperparameter configurations using the sweep feature of the Weights and Biases library.^40^ These optimization steps were performed using computing resources from the Argonne Leadership Computing Facility (ALCF), Illinois, USA.^41^ The computational runs were further facilitated via the DeepSpeed library^42^ and mixed precision training.^43^ The different ranges and distributions of values trialed for these runs can be seen in Table 3, with each data set being designated a full sweep across those hyperparameter ranges. Figure 5 shows the values trialed across the different hyperparameter ranges during the sweeps for the BERT-base model fine-tuned on the three QA data sets used. As mentioned earlier, the best-performing language model from each sweep−data set pair was chosen to represent the efficacy of the QA data set by testing on the hand-crafted thermoelectric test data set.

**Table 3:** Hyperparameter Space Explored in the Weights and Biases Sweeps

Hyperparameter	Minimum	Maximum	Distribution
Learning rate	10^–3^	10^–5^	Log-uniform (values)
Number of epochs	1	20	Integer uniform
Per device batch size	1	16	Integer uniform
Warm-up ratio	0.05	0.2	Uniform

**Figure 5:**
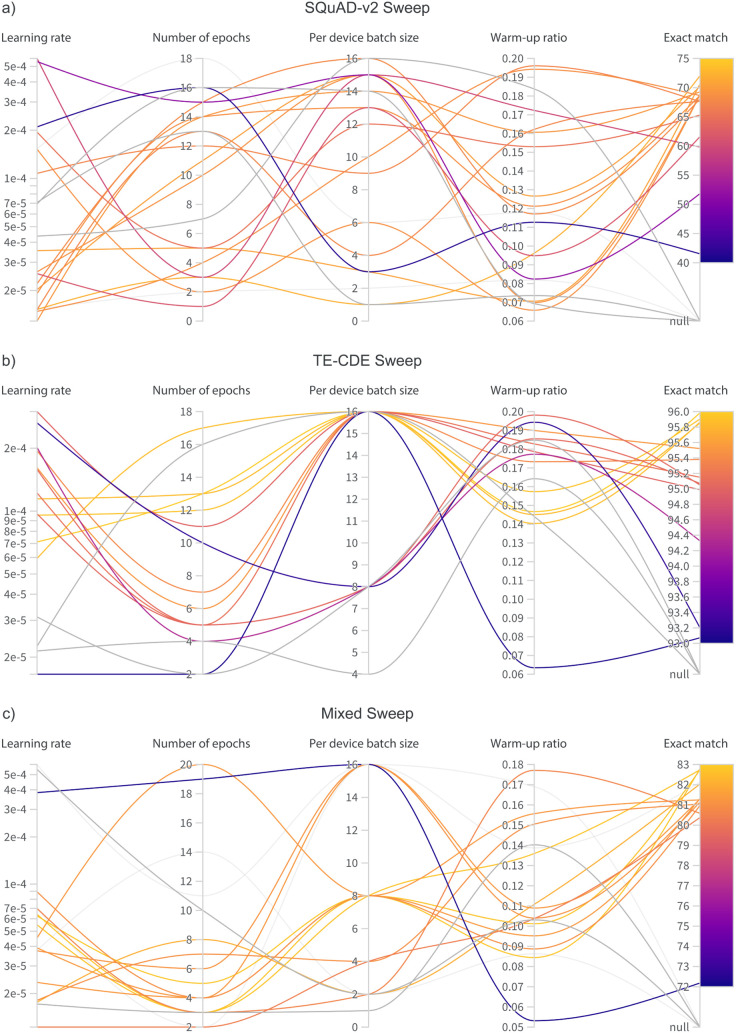
Visualization of the hyperparameter sets trialed and their corresponding exact-match score for the three QA data sets used. Visualization generated via Weights and Biases^40^ based on original experimental results.

Each sweep employed four compute nodes on the ALCF supercomputer, Polaris. Each node further controls four NVIDIA A100 GPUs, for a total of 16 GPUs utilized per sweep. The resource allocation requests, made via the Portable Batch System (PBS), queued for about one to 10 h and then each ran for about 8 h. Each sweep was run for approximately 16 h in total. This time-range allowed the Bayesian optimization technique to adequately explore the hyperparameter space and for the models to score highly on the exact-match validation metric. Exact match refers to the percentage of answers that are returned by the model matching perfectly with the expected answer. All the best-performing runs achieved an exact-match score above 70%, indicating that the model has adapted well to the QA data set task. An entire sweep was dedicated to every QA data set. As shown in Table 3, the hyperparameters queried were the per-device batch size, learning rate, number of epochs, and warm-up ratio. The per-device batch size of 1–16 results in a total batch size of 16–256, which covers a wide range of commonly used batch sizes in deep learning. The learning rate similarly trials a decent range between small and large learning rates according to a logarithmic uniform distribution. The number of epochs allows the sweep to explore whether longer training, going up to 20 times over the entire training data set, affords better performance or not. Finally, the warm-up ratio sets the relationship between how many steps are taken to “warm up” to the full target learning rate, based on the estimated number of steps taken to complete the training. The method of using a smaller learning rate and building up to higher rates has been shown to improve convergence in transformer models such as BERT.^44^ Figure 6 shows the exact-match scores for different runs, sorted by order, for the three different sweeps, as the Bayesian optimization method trials and discovers better hyperparameter combinations for the three QA data sets, with the TE-CDE-QA dataset showcasing clearly the improvements afforded by the sweep procedure. The other two QA data sets do not show such evident improvements, but this can be attributed to a lucky starting guess for the set of hyperparameters. Regardless, running the sweep allows confidence in the set of hyperparameters used as being quite optimal within the ranges explored. This process allowed the selection of a best-performing language model to represent each QA data set during the final testing.

**Figure 6:**
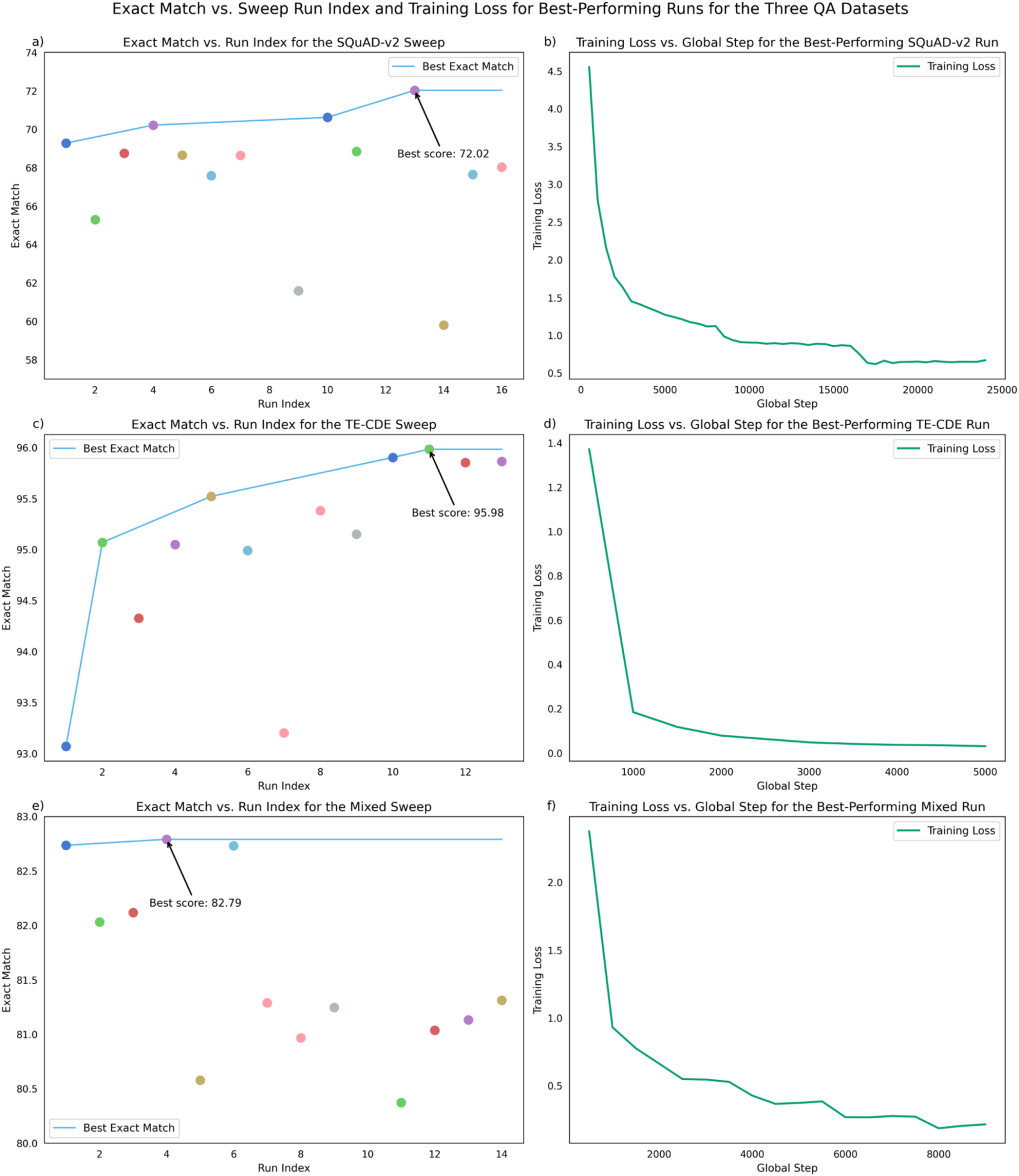
Plots (a c, and e) show the exact match versus run index for the three different QA-data set sweeps, with distinct color markers indicating runs with different hyperparameter sets and the azure line tracking the best performance. Plots (b, d, and f) showcase the training loss against the global training step for the best-performing model fine-tuned on the corresponding QA-data sets.

### Evaluation Metrics and Method

The evaluation of the different fine-tuned language models on the QA data sets was performed using the standard QA metrics of exact match and *F*1 score. These metrics were also further distinguished between the whole set of questions as well as answerable and unanswerable questions, according to the SQuAD-v2 paradigm. Exact match was defined in the previous section, while the *F*1 score is the harmonic mean between precision and recall. The *F*1 score attempts to describe the overall quality of the model predictions, balancing the ability of the model to capture correct answers while ignoring questions with no valid answers.

As already discussed, language models with different hyperparameters were fine-tuned on the three QA data sets using Weights and Biases sweeps. The best-performing model on the exact-match metric of its validation set was chosen to represent the efficacy of the data set. These models were then evaluated on the metrics mentioned above on the thermoelectric materials QA test data set that we algorithmically generated from the human-labeled annotations created using BRAT, in order to gauge the efficacy of the training data sets relative to the downstream task of information extraction. The three best-performing models fine-tuned on the three different data sets have been made available online.^45^

## Results and Discussion

### Technical Validation and Testing

The hyperparameters that afforded the best-performing BERT model on each QA data set, according to the exact-match metric, can be seen in Table 4. It should be noted that these exact-match scores refer to the respective validation portion of each QA data set, with the testing being performed later on the thermoelectric test data set. Every best-performing BERT model achieved an exact match of >70% on its validation set, which implies that the models are capable of adapting their weights to the QA data set task. The best-performing model fine-tuned on the mixed QA data set, scored 67.93% when evaluated on the thermoelectric materials test data set, outperforming the two best-performing BERT models fine-tuned on the TE-CDE-QA data set or SQuAD-v2, which scored 65.39% and 57.60%, respectively.

**Table 4:** Hyperparameter Values for the Best-Performing BERT Models that Have Been Fine-Tuned for Downstream Extractive QA Tasks

		Training QA data set	
Hyperparameter	SQuAD-v2	TE-CDE-QA	Mixed
Learning rate	1.52× 10^–5^	7.11× 10^–5^	6.26 × 10^–5^
Number of epochs	3	13	5
*Total* batch size	4	64	32
Warm up ratio	0.096	0.15	0.084
Exact-match validation	72.02%	95.98%	82.79%

Figure 7 shows the scores of these best-performing BERT models when evaluated on the thermoelectric test data set, across the studied metrics. The results indicate that the act of mixing both the QA data sets with different domain specificities can afford better performance on domain-specific downstream tasks, such as information extraction in the thermoelectric materials domain. The two QA data sets used in mixing differ: not only in domain-specificity, with SQuAD-v2 sourcing paragraph contexts comprising generic English language from different articles on Wikipedia, while the TE-CDE-QA data set specializes on the thermoelectric materials literature, as well as in context scope. Owing to the restrictions imposed on retrieving the location of the answer from its context, the TE-CDE-QA data set was limited to sentence-wide contexts, while SQuAD-v2 offers paragraph-wide contexts. Therefore, SQuAD-v2 can be characterized as being both semantically and syntactically broader in scope than the TE-CDE-QA data set. The last distinction stems from the fact that SQuAD-v2 was annotated by Amazon Mechanical Turks, while the TE-CDE-QA data set was algorithmically created from a thermoelectric materials-science database, which itself was automatically generated from the scientific literature using ChemDataExtractor. Regardless, the TE-CDE-QA data set outperforms SQuAD-v2, which can largely be attributed to domain and task-specificity.

**Figure 7:**
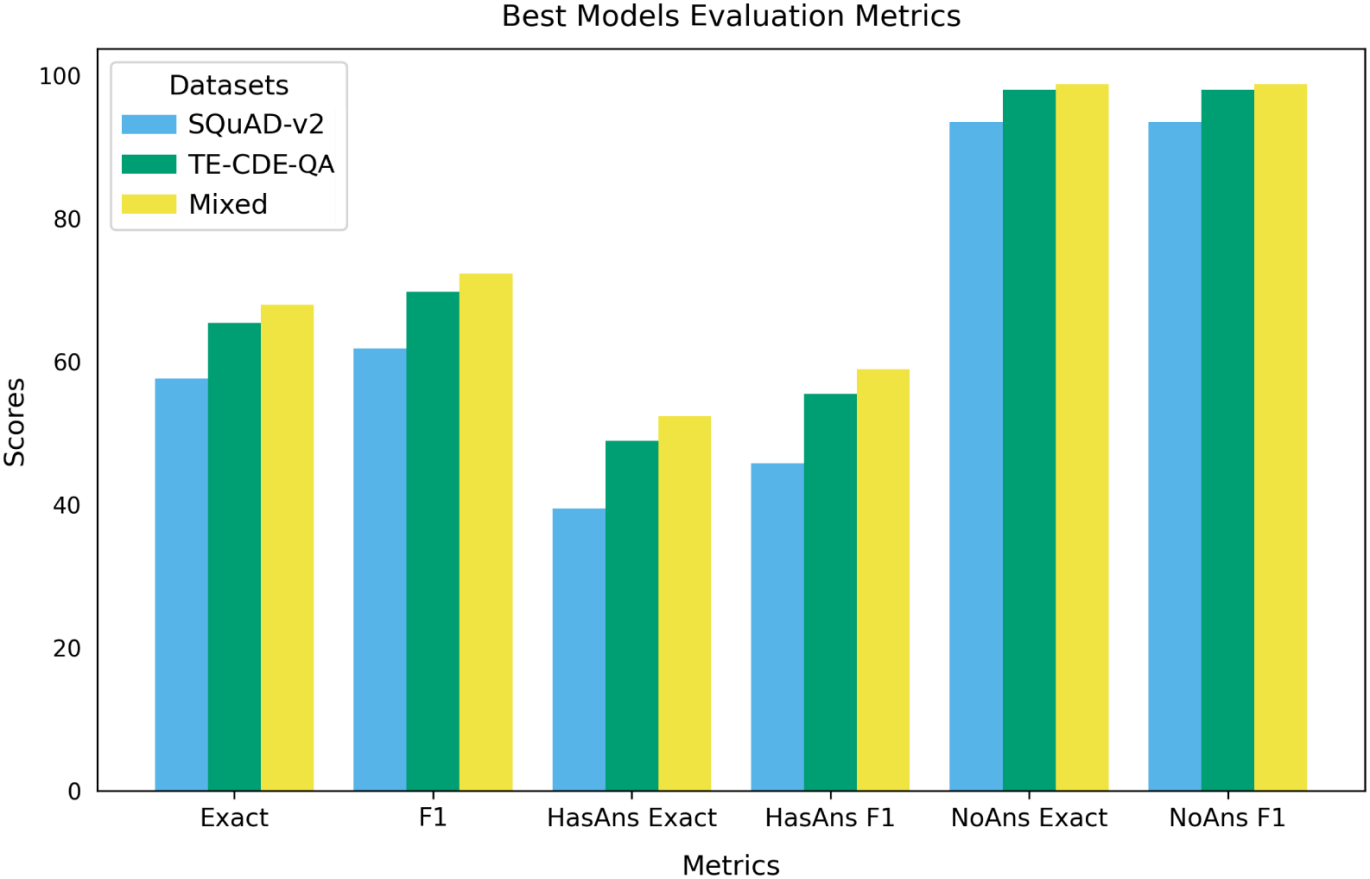
Comparison of the best-performing BERT models fine-tuned on three different QA data sets and evaluated on the thermoelectric materials QA test data set (labeled “HasAns”) and questions with no answers (labeled “NoAns”).

The best-performing BERT model fine-tuned on the mixed QA data set is superior on every metric, as seen in Figure 7 across both the exact match and *F*1 score, for all three categories: all questions, questions with answers, and unanswerable questions. The use of the TE-CDE-QA-fine-tuned model over the SQuAD-v2-fine-tuned model shows a significant improvement as well. This performance improvement can be largely attributed to the fact that the TE-CDE-QA data set is highly aligned with the thermoelectric test data set, underlying the benefits of utilizing domain-specific and task-specific data when training a model. Moreover, since the mixed QA data set performs even better than either the generic English-language-tailored SQuAD-v2 or the highly specialized TE-CDE-QA data set, this indicates that there is merit in combining different QA data sets of similar sizes (i.e., balanced data sets) with complementary strengths, such as semantics and syntax, in order to extend the capacity of a BERT model on a task that is more complex than any of the individual QA data sets.

Language models that have been fine-tuned on specific domains tend to perform poorly when evaluated on different domains.^46^ This section briefly investigates the performance degradation expected when evaluating the best-performing BERT models fine-tuned on the two QA new data sets, TE-CDE-QA, and the mixed data set, on the validation set from SQuAD-v2. As a point of comparison, the best-performing SQuAD-v2-fine-tuned BERT model scored 72.02% on the exact-match metric on the same validation set. Interestingly, the mixed QA data set scores a competitive 72.09%, which indicates that while the BERT model has improved on the specific thermoelectric domain by leveraging learning from both data sets, its weights have adapted in such a way that the learning afforded from SQuAD-v2 persists when applied to a generic English-language QA task, such as SQuAD-v2. This echoes a result similar to the one seen in the development of a BatteryBERT model,^4^ where that model further pretrained on a battery materials corpus outperforms the original BERT-base model on the SQuAD-v1 data set.^12^ The result here is similar, but in the realm of fine-tuning, showcasing the ability of BERT models to sometimes adapt to a specialized domain without their performance being significantly degraded on generic English-language contexts. On the contrary, the BERT model that was fine-tuned exclusively on TE-CDE-QA data set, as expected, performs particularly poorly on the SQuAD-v2 task, scoring 35.04% on the exact-match metric, since it had not been trained on any generic English-language or even paragraph-wide contexts. Moreover, when evaluating the thermoelectric subset within the validation set of the mixed model, an exact-match score of 95.54% was observed; this is nearly identical to the 95.98% exact-match score for the TE-CDE-QA model seen in Table 4. This underlines the ability of the mixed QA model to perform well at both in-domain semantics and complex syntax. As a result, it matches the TE-CDE-QA model on pure domain QA and outperforms it on the test set when both domain knowledge and complex syntactic understanding are required.

### Discussion

As seen in Table 4, there are relative asymmetries between the hyperparameters that afford the best-performing BERT model for each QA data set, as well as the exact-match scores that were achieved on their respective *validation* sets. It is reasonable to consider that the “easiest” QA data set for the BERT model to learn is the TE-CDE-QA data set. Despite its semantic specificity, this data set reflects a sentence-wide QA task, such as the one performed by ChemDataExtractor, which can be expected to be less demanding than extracting answers from general human-written questions on lengthier paragraph-wide contexts. This is further corroborated by the very high exact-match score that the BERT models achieve on the validation portion of the TE-CDE-QA data set. This demonstrates the capacity for the BERT model to learn how ChemDataExtractor extracts information when adapted to the thermoelectric materials domain. As can be seen in Figure 6b, the exact-match score for the TE-CDE-QA validation data set starts at >93%, even with the first set of hyperparameters trialed, and rises up to almost 96%. The other best-performing BERT models achieve an exact-match score of 72.02% and 82.79%, for the mixed QA data set and SQuAD-v2, respectively. Naturally, the exact-match validation score of the BERT model fine-tuned on the mixed QA data set sits between the “easier” TE-CDE-QA and the “more challenging” SQUAD-v2.

There is a wide range between the best hyperparameter sets discovered during the sweeps for the three different QA data sets. This implies that the scores of the different optimized BERT models, when evaluated on the thermoelectric test QA data set, have more to do with the efficacy of how the QA data sets lend themselves to the fine-tuning of a BERT model for a QA task, rather than a fortuitous selection of hyperparameters. It should be noted that only four hyperparameters have been queried for this kind of optimization, which may leave room for further improvements, such as using different kinds of optimization algorithms that update the model weights, weight decays, or others.

The data presented in Table 4 and Figure 5 imply that simpler sentences may generally benefit from fine-tuning over a greater number of epochs, while complex passages can be fine-tuned with fewer epochs. Previous work that explores BERT fine-tuning options concludes that 2–4 epochs are suitable for most QA tasks, model instabilities occur when trained on highly varied or noisy data, and domain similarity governs the optimal BERT fine-tuning schedule.^17,47,48^ However, to our knowledge, no study has systematically explored how textual complexity modulates the optimal number of epochs independently of domain shift. The patterns that we observe may reflect faster overfitting on linguistically complex passages versus more consistent gradient signals when BERT models are fine-tuned on QA data sets containing simpler text. While our data sets are limited, this preliminary finding motivates future work to explore and quantify the effect of text complexity (e.g., via perplexity or syntactic depth) and potentially inform complexity-aware early stopping fine-tuning schedules.

Figure 8 illustrates a case where the best-performing BERT model fine-tuned on the mixed QA data set outdoes the other best-performing BERT models which were fine-tuned on the TE-CDE-QA data set or SQuAD-v2. The given context contains two full thermoelectric *ZT* records, with information for one of them spanning multiple sentences. Relating information across two different spans reflects a known shortfall of the thermoelectric-bespoke version of ChemDataExtractor used in previous work.^18^ Since the TE-CDE-QA data set was created from a thermoelectric materials database^18^ which was in turn generated by this adaptation of ChemDataExtractor, it is natural to expect that the respectively fine-tuned BERT model might falter, as seen in the example shown in Figure 8. Conversely, the best-performing SQuAD-v2-fine-tuned BERT model seemed to struggle with identifying the correct answers entirely in this example, perhaps owing to a lack of data with thermoelectric contexts encountered during the fine-tuning process. Finally, the BERT model fine-tuned on the mixed QA data set demonstrated both accurate semantic and syntactic capabilities at answering the questions. Naturally, not all examples in the test set reflect this exact situation, but the observed trend is that SQuAD-v2 exhibits the best capacity at extracting answers that span multiple sentences, because of its paragraph-wide training, but lacks the ability afforded by our TE-CDE-QA data set to correctly treat thermoelectric-specific nomenclature. The mixed QA data set appears to successfully combine both aspects. All models leverage the ability to process English language to an extent, thanks to the pretraining of the original BERT model on the BookCorpus and English Wikipedia.^17^ Although we used the generic English-encoded BERT-base model to maintain a consistent baseline across QA benchmarks, it is possible that other materials-specific checkpoints (e.g., MatBERT,^50^ MatSciBERT^3^ could alternatively be used as initialization points to afford similarly specialized models, further fine-tuned on the TE-CDE-QA or mixed QA data set. Such models might even yield gains in the performance of models presented in this study, as they are able to leverage this benefit of prior exposure to materials-science terminology and associated constructs.

**Figure 8:**
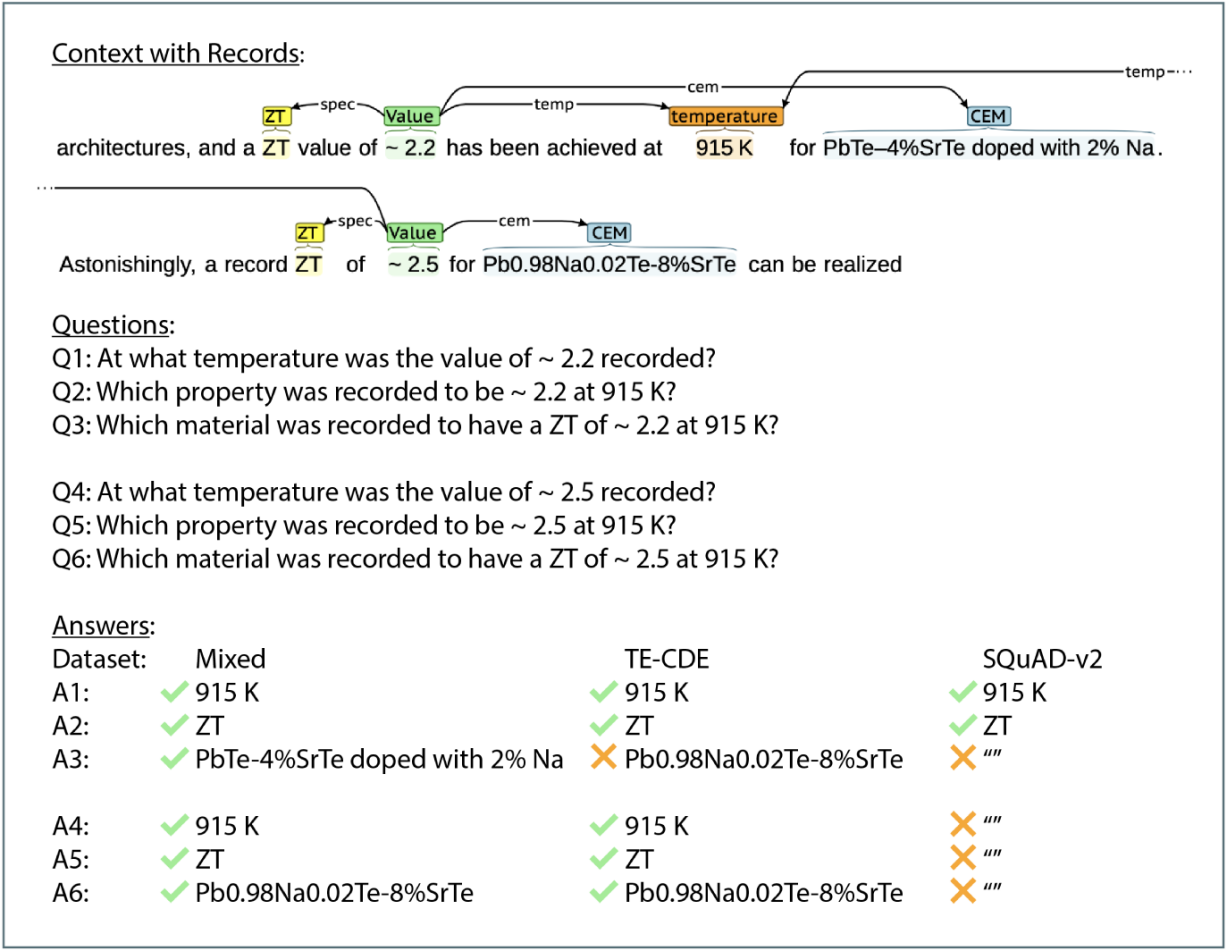
An example showcasing the improved performance of the BERT model fine-tuned on the mixed QA data set. The empty quotation marks refer to no answers being returned by a BERT model. The annotations were made using the BRAT rapid annotation tool^19^ on text reproduced under the terms of the Creative Commons CC BY-NC-ND license from ref. 49: Qian et al. 2018, Elsevier.

We also investigated where our models look during inference. Thereby, we started with our best-scoring model (fine-tuned on the mixed QA data set) and we averaged the attention flows across heads in the last three layers, to produce a question-to-context attention map. For sake of presentation, and without degrading our argument, we used a simplified example:

“At 290 K, the value of power factor for Ca_3_SnO is 160 W m^–1^K^–2^”,

and then asked the following three standard questions while observing the attention maps:

(1) “At what temperature was the value of 160 W m^–1^K^–2^ recorded?”
  - Figure 9a, top left, shows that the ‘temperature” token attends most strongly on “290” and “K,” correctly linking the concept of property to its respective measurement.
  - Figure 9a, upper center-left, reveals that attention from “the value of” peaks strongly to “power factor,” showing the model’s ability to map abstract phrasing to domain terminology.
(2) Figure 9b, top center-left, illustrates how “Which property was recorded to be 160 W m^–1^K^–2^ at 290 K?”
  - “Which property” attends most strongly to “power factor,” confirming correct property identification.
(3) “Which material was recorded to have a power factor of 160 W m^–1^K^–2^ at 290 K?”
  - Despite the way that the chemical formula “Ca_3_SnO” is split across multiple tokens, “which material” strongly focuses attention on the start and end token of the material name, as displayed in Figure 9c, top and bottom, at the two central bars.

**Figure 9:**
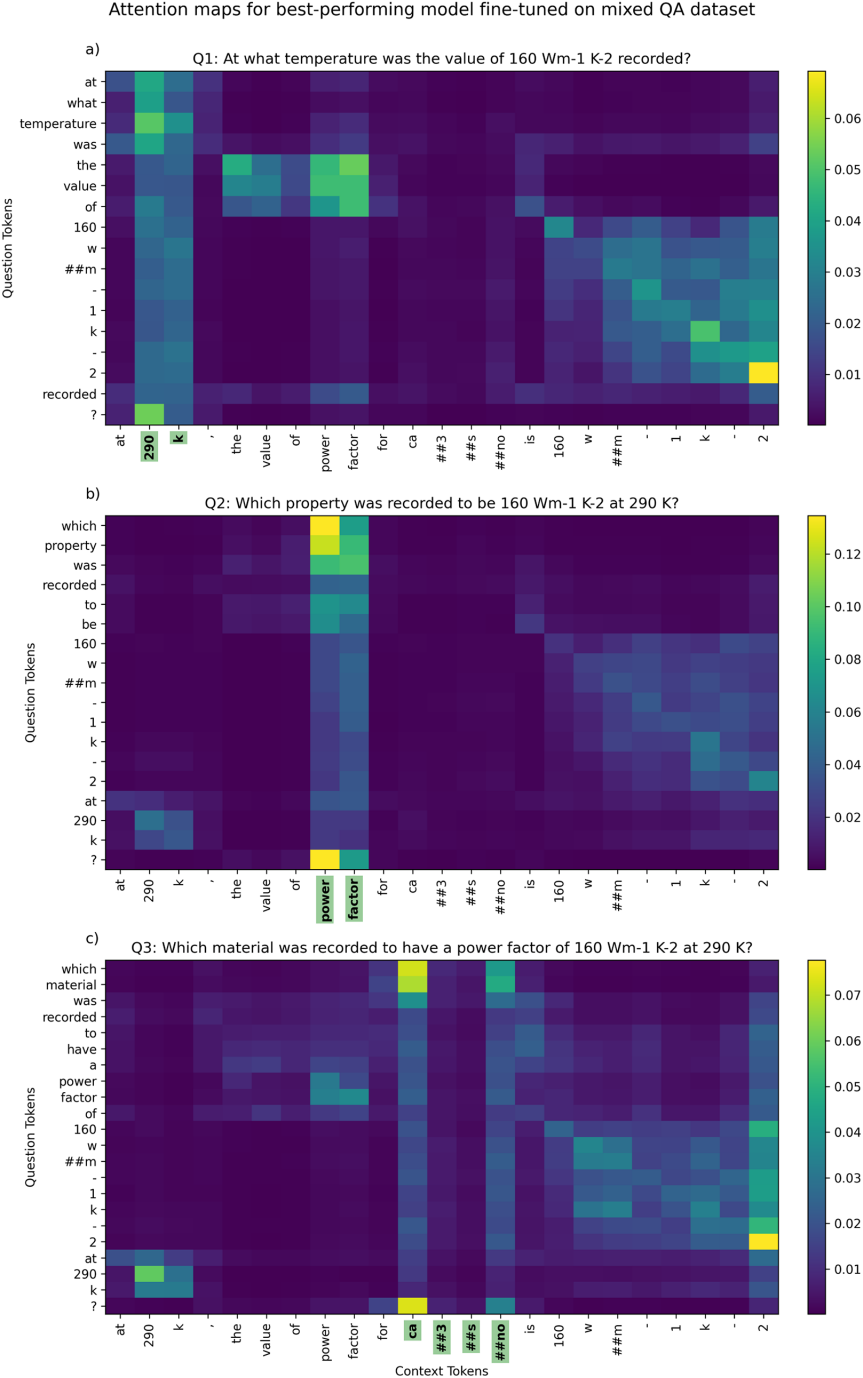
Attention heatmaps between in-domain questions and example context for the best-performing model which had been fine-tuned on the mixed QA data set. The *x*-axis labels highlighted in green show the answers returned by the model (which are correct for all questions in this example).

When comparing these heatmaps in Figure 9 against the other best-performing models that had been fine-tuned on TE-CDE-QA or SQuAD-v2 (Figures S1–S3), the mixed and TE-CDE-QA models show similar, well-aligned attention patterns, whereas the SQuAD-v2 model’s attention seems less well-aligned (e.g., it fails to link “property” to “power factor” in question 2 and erroneously returns the material as an answer). These heatmaps suggest that domain-specialized fine-tuning not only boosts accuracy but also improves the attention of BERT models on relevant spans. We acknowledge, however, that attention is not a definitive explanation of model decisions,^51^ and that future work could couple this type of inquiry with gradient-based analyses.^52,53^

A possible limitation of this research lies in the very high scores achieved by all three BERT models in the unanswerable questions category, seen at >90%. This shows that these models become very good at not returning an answer when no answer should be returned, implying that the algorithm generating the unanswerable questions might have room for improvement. Instead of picking an unfindable data point of the correct type from the thermoelectric materials database,^18^ a better approach might be to use a text span contained within the context which an information extraction tool might mistakenly identify as an answer to a preceding question. By creating more challenging unanswerable questions, this will afford better QA models. This approach may be examined in future work. Furthermore, while the BERT models trained on the TE-CDE-QA data set demonstrate the ability to perform extractions beyond the strict scope of ChemDataExtractor that was used to create the thermoelectric materials source database,^18^ from which the QA data set was crafted, they still showcase similar limitations, as expected. These intrinsic limitations of the thermoelectric-bespoke adaptation of ChemDataExtractor have been discussed in more detail in previous work.^18^ The mixing of the TE-CDE-QA data set with SQuAD-v2 ameliorates these issues, on which the improved performance can be partly attributed.

## Conclusions

This work has investigated the efficacy of autogenerating a large QA data set from a domain-specific materials database and using it to fine-tune BERT models to perform information extraction in the field of thermoelectric materials. It also investigated the benefits of combining more domain-specific data sets with shorter contexts, such as the generated TE-CDE-QA data set, with more generic data sets with larger contexts, such as SQuAD-v2. The research showed that BERT models can afford better performance on a thermoelectric materials test data set, generated from human-labeled data in the field, for an extractive QA task across temperatures, specifiers of the five seminal thermoelectric material properties (*ZT*, Seebeck coefficient, thermal and electrical conductivity, power factor), and material names, when trained on an in-domain QA data set. We further showed that this performance can be increased by combining different QA data sets of a similar size (i.e., they are balanced), but which vary in terms of syntactic and semantic scope, with the mixed QA data set delivering the highest scores on the final test data set.

Perhaps surprisingly, it was shown that BERT models that have been fine-tuned on in-domain QA data sets can outperform models fine-tuned on nonspecialized QA data sets, even if the in-domain data have limited context lengths or are less accurate than the nonspecialized QA data set. Finally, it was shown that there is potential for conducting relationship extraction using BERT-like language models, by fine-tuning them on large QA data sets that have been autogenerated from existing materials-science databases. The scores achieved by the best-performing BERT model fine-tuned on the mixed QA data set also indicate that even for challenging tasks such as scientific information extraction, SLMs, (here, 110 M parameters) of discriminative nature can deliver satisfactory results. These BERT models were only fine-tuned on the QA data sets, which is much less computationally intensive compared to pretraining, especially if pretraining from scratch. This underlines the usefulness of automatically crafting or generating large domain-specific QA data sets to further empower the application of language models in the scientific domain. Indeed, there is an urgent need for new ways to apply SLMs and with minimal modification, from an energy-sustainability perspective. This need is noted given the high energy overheads that are required for pretraining language models. Even for an SLM, such as BERT-base which was applied in this study, the computational load required to pretrain it is estimated to afford 1435 lbs of *CO*_2_ emissions; for comparison, this is similar to the amount of *CO*_2_ emitted per passenger on a flight from New York to San Francisco (1984 lbs).^54^

The results were first established by using hyperparameter optimization across learning rate, batch size, number of epochs, and warm-up ratio. The sweeps showed that a variety of hyperparameter sets achieve close to the maximum score, implying that it was relatively easy for the BERT models to adapt to these QA data sets. Future work could examine whether the approach of generating QA data sets from existing materials-science databases affords better results on domain-specific test data sets, and if the trend of improved performance after mixing QA data sets with different syntactic and semantic scopes persists across domains. The scores themselves are good but still offer room for improvement, which could be achieved through the crafting of better fine-tuning QA data sets, using different model architectures, or different training approaches.

## Supplementary Material



## Data Availability

The scripts used to generate the thermoelectric test data set from the BRAT annotation files and those used to generate the TE-CDE-QA data set from our thermoelectric materials database^18^ are available online.^21,23^ The thermoelectric QA data sets used for training and validation, and the thermoelectric QA test data set used for testing the fine-tuned BERT models have also been made available online.^22,38^ The three best-performing fine-tuned BERT models on the different QA data sets have been uploaded to the Molecular Engineering Group area of Huggingface.^45^

## Notes

Details on data sets and hyperparameters used during fine-tuning optimization and test results for the best-performing BERT models (PDF)
