# Supplementary material for: Autogenerating a Domain-Specific Question-Answering Data Set from a Thermoelectric Materials Database to Enable High-Performing BERT Models
Source: J Chem Inf Model. 2025 Aug 7;65(16):8579–92. doi: 10.1021/acs.jcim.5c00840 (PMC12381847; doi:10.1021/acs.jcim.5c00840)
Supplement: Supplementary file 1 [file ci5c00840_si_001.pdf]

Auto-generating a domain-specific question-answering dataset from a thermoelectric materials database to enable high-performing BERT models

Odysseas Sierepeklis[1],
Jacqueline M. Cole[1,2]

[1]Cavendish Laboratory, University of Cambridge,
J. J. Thomson Avenue, Cambridge, CB3 0HE, UK
[2]Science and Technology Facilities Council, Rutherford Appleton Laboratory,
Harwell Science and Innovation Campus, Didcot, Oxfordshire, OX11 0QX, UK

jmc61@cam.ac.uk

Supporting Information

Hyperparameter-optimized BERT Evaluations

Table S1: Exact match and $F1$ score from the thermoelectrics QA test dataset evaluation for the models fine-tuned on the three different QA datasets, distinguishing between all questions, questions with answers (HasAns), and questions without answers (NoAns).

**Evaluation Metric**	**Training QA dataset**		
	SQuAD-v2	TE-CDE-QA	Mixed
Exact match	57.60%	65.39%	67.93%
$F1$	61.82%	69.78%	72.29%
HasAns exact match	39.47%	48.92%	52.35%
HasAns $F1$	45.81%	55.53%	58.92%
NoAns exact match	93.54%	98.03%	98.80%

Attention Heatmaps

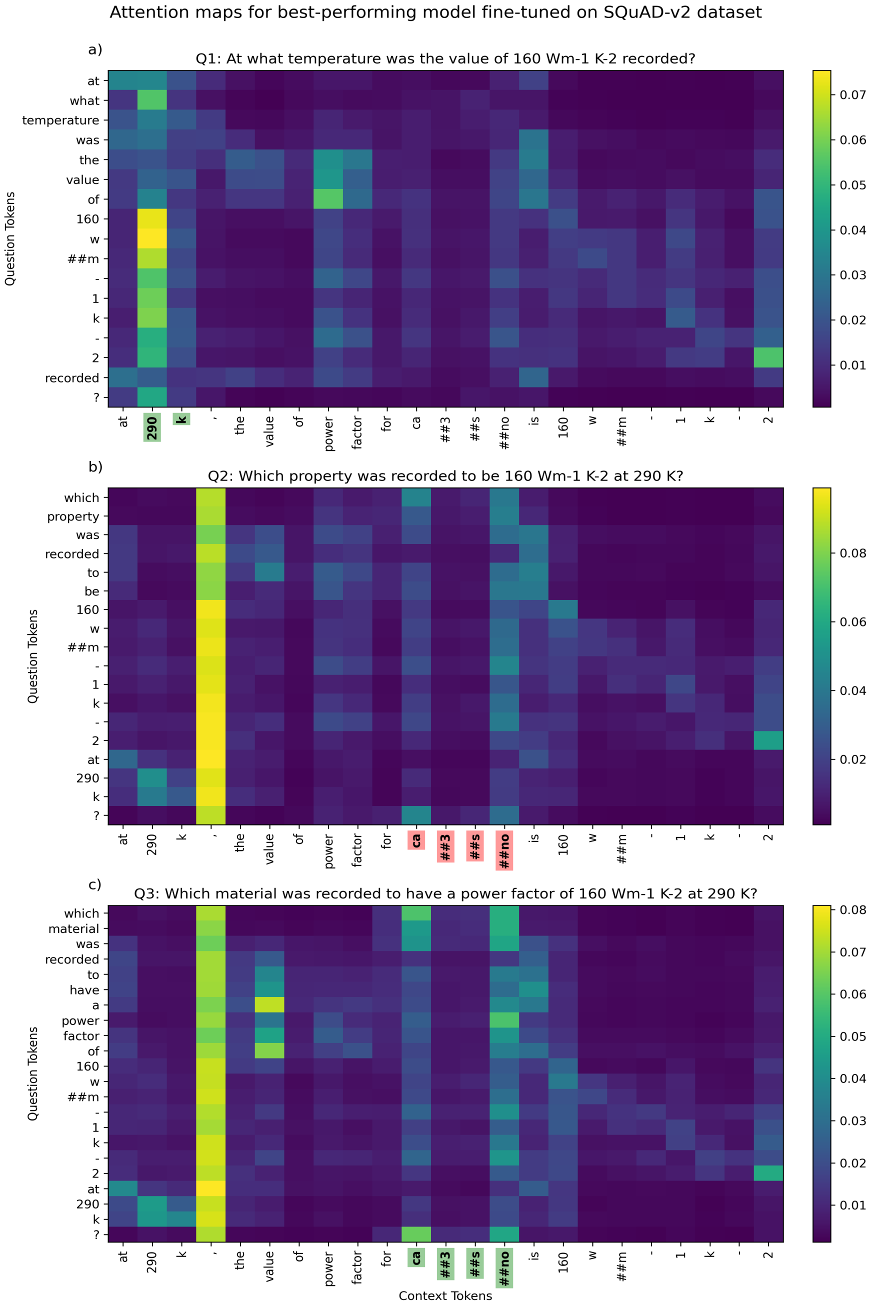

Figure S1: Attention heatmaps between in-domain questions and example context for the best-performing model fine-tuned on SQuAD-v2. The highlighted x-axis labels show the answer returned by the model (green if correct, red if wrong).

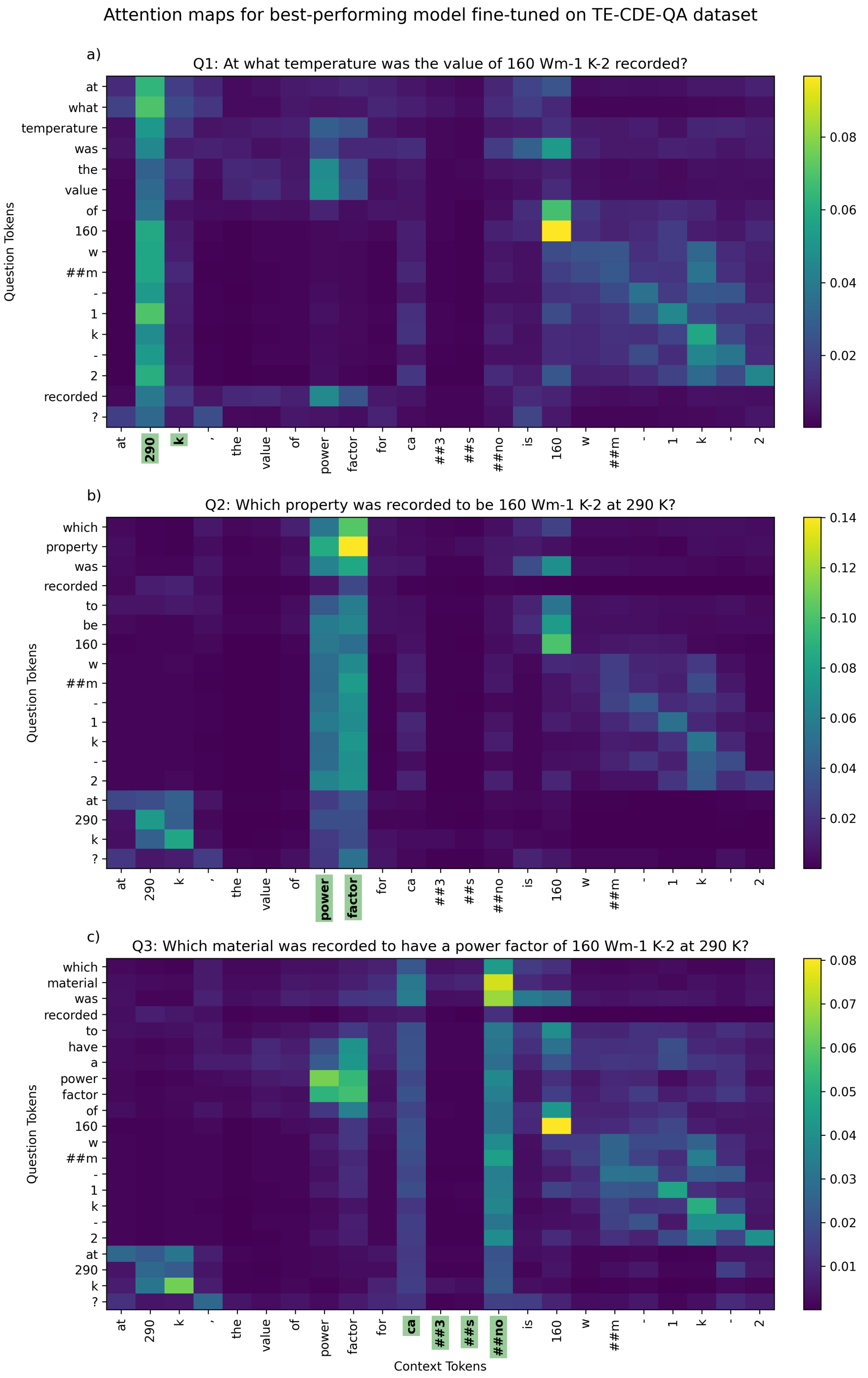

Figure S2: Attention heatmaps between in-domain questions and example context for the best-performing model fine-tuned on the TE-CDE-QA dataset. The highlighted x-axis labels show the answer returned by the model (green if correct, red if wrong).

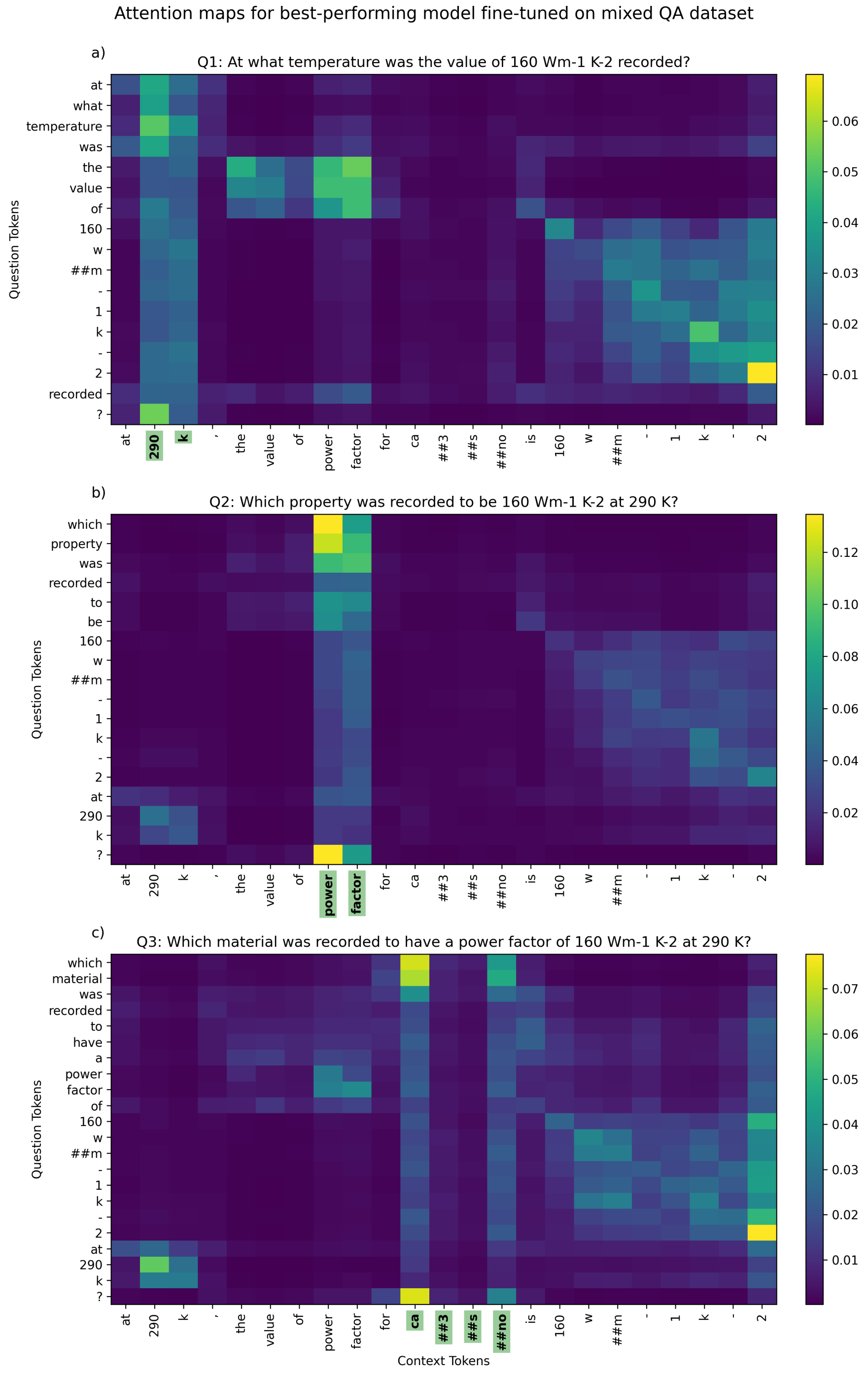

Figure S3: Attention heatmaps between in-domain questions and example context for the best-performing model fine-tuned on the mixed QA dataset. The highlighted x-axis labels show the answer returned by the model (green if correct, red if wrong).
